# Attainment and characteristics of clinical remission according to the new ACR-EULAR criteria in abatacept-treated patients with early rheumatoid arthritis: new analyses from the Abatacept study to Gauge Remission and joint damage progression in methotrexate (MTX)-naive patients with Early Erosive rheumatoid arthritis (AGREE)

**DOI:** 10.1186/s13075-015-0671-9

**Published:** 2015-06-11

**Authors:** Josef S. Smolen, Jürgen Wollenhaupt, Juan J. Gomez-Reino, Walter Grassi, Corine Gaillez, Coralie Poncet, Manuela Le Bars, Rene Westhovens

**Affiliations:** Division of Rheumatology, Department of Medicine 3, Medical University of Vienna, and 2nd Department of Medicine, Hietzing Hospital, Waehringer Guertel 18-20, Vienna, A-1090 Austria; Schön-Klinik, Hamburg-Eilbek, Hamburg, Germany; Hospital Clinico Universitario, Santiago de Compostela, Spain; Clinica Reumatologica, Università Politecnica delle Marche, Ancona, Italy; Bristol-Myers Squibb, Rueil-Malmaison, France; Docs International, Nanterre, France; Skeletal Biology and Engineering Research Center, Department of Development and Regeneration KU Leuven; Rheumatology, University Hospitals Leuven, Leuven, Belgium

## Abstract

**Introduction:**

This study evaluated various remission criteria in abatacept plus methotrexate (MTX)-treated patients with early rheumatoid arthritis (RA). We aimed to investigate the time to, and sustainability of, remission, and to evaluate the relationship between remission, function and structure.

**Methods:**

Post hoc analyses were performed from the 12-month, double-blind period of the Abatacept study to Gauge Remission and joint damage progression in methotrexate (MTX)-naive patients with Early Erosive rheumatoid arthritis (AGREE) in patients with early RA (≤2 years) and poor prognostic factors, comparing abatacept plus MTX (n = 210) versus MTX alone (n = 209).

**Results:**

At month 12, Disease Activity Score 28, Simplified Disease Activity Index (SDAI), Clinical Disease Activity Index and Boolean remission rates were, for abatacept plus MTX versus MTX alone: 47.6 % versus 27.3 %, 33.3 % versus 12.4 %, 34.3 % versus 16.3 %, and 23.8 % versus 5.7 %, respectively. Cumulative probability demonstrated higher proportions achieving first remission and first sustained remission for abatacept plus MTX versus MTX alone (e.g., 23.3 % [95 % confidence interval (CI): 17.6, 29.1] vs 12.9 % [8.4, 17.5] for first SDAI remission over 0–6 months). For patients in SDAI remission at month 3, mean Health Assessment Questionnaire-Disability Index at month 12 was 0.20 versus 0.50 for abatacept plus MTX versus MTX alone. Mean changes in radiographic score from baseline to month 12 were minimal for patients in SDAI remission at month 3 in both groups, while less structural damage progression was seen, 0.75 versus 1.35, respectively, for abatacept plus MTX versus MTX alone for patients with moderate/high disease activity at month 3 (adjusted mean treatment difference: −0.60 [95 % CI: −1.11, −0.09; *P* < 0.05]).

**Conclusions:**

High proportions of abatacept plus MTX-treated patients achieved stringent remission criteria. Remission was associated with long-term functional benefit; dissociation was seen between clinical and structural outcomes for abatacept. These findings highlight the impact of reaching stringent remission targets early, on physical function and structural damage, in MTX-naïve biologic-treated patients.

**Trial registration:**

ClinicalTrials.gov identifier NCT00122382. Registered 19 July 2005.

**Electronic supplementary material:**

The online version of this article (doi:10.1186/s13075-015-0671-9) contains supplementary material, which is available to authorized users.

## Introduction

According to the treat-to-target principle, patients with rheumatoid arthritis (RA) should be monitored closely, with the goal of achieving clinical remission or low disease activity (LDA) within 6 months [1, 2], and then regularly assessed for sustained remission [3]. Various criteria have been used to formally define remission in clinical trials, of which the Disease Activity Score (DAS)28 (score of <2.6) has traditionally been used most frequently. However, patients in DAS28 remission may experience residual disease activity, as evidenced by swollen joint count (SJC) in particular [4–7], which can lead to irreversible structural damage [8, 9]. American College of Rheumatology (ACR)-European League Against Rheumatism (EULAR) recommendations [2] advocate using more stringent remission criteria, namely the Simplified Disease Activity Index (SDAI) and a Boolean definition. Alternative definitions proposed for use in clinical practice, where measurement of acute-phase reactants is often unavailable, are the Clinical Disease Activity Index (CDAI) and a corresponding Boolean definition.

Abatacept is a biologic disease-modifying antirheumatic drug (DMARD) that selectively modulates T cell co-stimulation [10] and has shown efficacy in several clinical trials, including the Abatacept study to Gauge Remission and joint damage progression in methotrexate (MTX)-naive patients with Early Erosive rheumatoid arthritis (AGREE) [10–12].

The main objective of the present analysis was to explore remission in patients with early RA treated with abatacept plus MTX in light of the current, stringent ACR-EULAR definitions of remission [2]. These new remission criteria are frequently believed to be difficult to achieve [13, 14]. Therefore, we evaluated patients with early RA who had been treated with either MTX alone or abatacept plus MTX in the AGREE trial. We also investigated sustainability of initial remission and evaluated the relationship of disease activity with function and structure in these patients. As will be demonstrated, a considerable number of patients can indeed achieve (and sustain) remission, even when using the new and stringent remission criteria.

## Methods

### Patient population and study design

This post hoc analysis is based on data from the previously published AGREE Phase III study (ClinicalTrials.gov identifier NCT00122382), in which patients were randomized (1:1) to receive abatacept plus MTX or MTX alone over a 12-month, double-blind period [15]. Patients were MTX naïve (or prior exposure was ≤10 mg/week for ≤3 months); had early RA (≤2 years); were positive for rheumatoid factor and/or anti-cyclic citrullinated peptide antibodies; had evidence of erosive changes on hands, wrists or feet; and had tender joint count (TJC) ≥12, SJC ≥10 and C-reactive protein (CRP) ≥4.5 mg/dL.

### Outcome measures

Individual core set variables [16] were assessed monthly. Although DAS28 (CRP) <2.6 (DAS28 remission) [17] at month 12 was a co-primary endpoint of the original study, in this post hoc analysis we calculated ACR-EULAR index-based remission rates, with remission defined as SDAI ≤3.3 and CDAI ≤2.8 (see Additional file 1: Table S1) [2, 4, 18, 19]. Remission according to the ACR-EULAR Boolean definitions (clinical practice with 28-joint counts: TJC28 ≤1, SJC28 ≤1 and patient global assessment (PGA) ≤1; clinical trials with 28-joint counts and laboratory measures: TJC28 ≤1, SJC28 ≤1, CRP ≤1 mg/dL and PGA ≤1; clinical practice with 66/68-joint counts: TJC68 ≤1, SJC66 ≤1 and PGA ≤1; or clinical trials with 66/68-joint counts and laboratory measures: TJC68 ≤1, SJC66 ≤1, PGA ≤1 and CRP ≤1 mg/dL) was also evaluated post hoc [2]. Level of disease activity was measured using DAS28 (CRP), SDAI and CDAI (low disease activity (LDA), SDAI >3.3–11, CDAI >2.8–10 or DAS28 ≤3.2; moderate disease activity (MDA), SDAI >11–26, CDAI >10–22, or DAS28 >3.2– ≤ 5.1; high disease activity (HDA), SDAI >26, CDAI >22, or DAS28 >5.1).

Functional disability was evaluated monthly using the Health Assessment Questionnaire-Disability Index (HAQ-DI). Structural damage progression was evaluated by radiographs at baseline and month 12, with changes over time evaluated according to the total Genant-modified Sharp score (TGSS) [20] and non-progression defined as a mean change in TGSS of ≤0.

### Statistical analyses

In the original study, DAS28 (CRP)-defined remission was evaluated for the intention-to-treat population, with patients who discontinued considered non-responders. For the purpose of this report, analyses were based on patients with DAS28 and SDAI data available at baseline, month 6 and month 12. Analyses were performed post hoc for patients who had received abatacept plus MTX or MTX alone in the double-blind period. The proportions of patients achieving remission and LDA according to DAS28 (CRP), SDAI and CDAI, and remission according to Boolean outcomes, were analyzed as point estimates with 95 % confidence intervals (CI). Analyses were performed to determine shifts in SDAI status (remission, LDA, MDA and HDA) from month 6 to 12, presented as the proportions of patients in each SDAI state at month 6 who achieved each SDAI state at month 12. Cumulative probability of time to achieve first remission/LDA and sustained first remission/LDA (defined as maintained at all subsequent visits up to month 12) according to DAS28, SDAI and CDAI were evaluated based on Kaplan–Meier estimation with 95 % CI. Patients who lost first remission/LDA status were censored at the time of loss.

The relationship between DAS28, SDAI and CDAI remission was investigated by determining the proportions (95 % CI) of patients in DAS28 remission who had also achieved SDAI or CDAI remission, and by evaluation of the median values of core set variables (TJC, SJC, PGA, evaluator’s global assessment (EGA), CRP, erythrocyte sedimentation rate (ESR)) for patients achieving remission. We also evaluated mean scores for core set variables for patients achieving remission at month 12 but not at month 6. Finally, we evaluated the relationship between disease activity at month 3 and functional and structural outcomes at month 12, including mean scores (SD) and mean changes (95 % CI) by disease activity status (remission, LDA or MDA and HDA pooled) at months 3 and 12. Mean changes, treatment differences and corresponding 95 % CI were adjusted based on an analysis of covariance model with treatment, baseline score and disease status as covariates.

## Results

### Patient disposition

In the original AGREE study, 253 and 256 patients were randomized to receive MTX alone and abatacept plus MTX, respectively; 227 and 232 patients, respectively, completed the 12-month, double-blind period [15]. Discontinuations were mainly due to adverse events (4.3 % in the MTX alone group vs 3.5 % in the abatacept plus MTX group) and lack of efficacy (3.2 % and 0 %, respectively). Included in this post hoc analysis were 209 patients receiving MTX alone and 210 receiving abatacept plus MTX, with DAS28 and SDAI data available at baseline, month 6 and month 12.

### Baseline demographics and clinical characteristics

Demographics and baseline clinical characteristics for the analysis population were comparable between groups (see Additional file 2: Table S2), and were similar to those reported previously for the overall study population [15] with mean disease duration of ≤7 months and high mean disease activity levels. In both treatment groups, patients attaining remission or LDA at month 12 generally had numerically lower baseline TJC, SJC and DAS28, SDAI and CDAI, as well as mean duration of RA, than patients with MDA or HDA at month 12 (see Additional file 3: Table S3).

### Remission outcomes

**Fig. 1:**
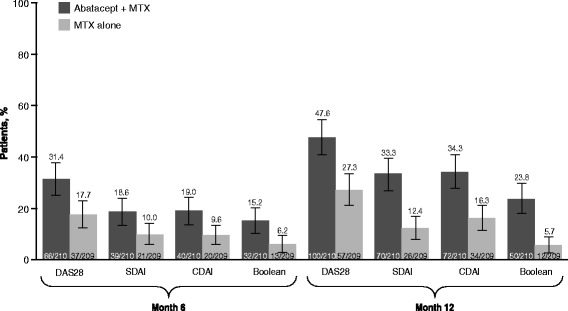
Overall remission rates at months 6 and 12 based on as-observed analyses for patients with data available at baseline, month 6 and month 12. Disease Activity Score 28 (*DAS28*) remission = DAS28 (C-reactive protein (CRP)) <2.6; Simplified Disease Activity Index (*SDAI*) remission = SDAI ≤3.3; Clinical Disease Activity Index (*CDAI*) remission = ≤2.8. *MTX* methotrexate

At month 12, remission was achieved in 27.3 % (95 % CI 21.2, 33.3) of patients receiving MTX alone compared with 47.6 % (95 % CI 40.9, 54.4) of patients receiving abatacept plus MTX by DAS28 criteria, by 12.4 % (95 % CI 8.0, 16.9) and 33.3 % (95 % CI 27.0, 39.7) by SDAI criteria, by 16.3 % (95 % CI 11.3, 21.3) and 34.3 % (95 % CI 27.9, 40.7) by CDAI criteria, and by 5.7 % (95 % CI 2.6, 8.9) and 23.8 % (95 % CI 18.0, 29.6) by Boolean criteria (the 95 % CI did not overlap) (Fig. 1). Thus, a large proportion of patients achieved remission according to the stringent SDAI, CDAI and Boolean criteria, although, as expected, fewer than by DAS28 (CRP) criteria. Indeed, one out of three patients achieved SDAI remission and almost 25 % of patients achieved Boolean-defined remission (28-joint count with laboratory measures) at month 12. Similar respective rates were seen for CDAI remission and for Boolean remission evaluated without CRP using 66/68-joint counts (see Additional file 4: Figure S1A). Also, large proportions of patients achieved LDA at month 12 with abatacept plus MTX and MTX alone, but – in contrast to the remission criteria – proportions were comparable regardless of the index used, although numerically higher with the combination (see Additional file 4: Figure S1B).

Remission rates at 6 months were lower but still considerable (Fig. 1). Indeed, for patients receiving abatacept plus MTX, remission rates increased by >50 % from months 6 to 12 with every index used; most patients reaching remission after 12 months were derived from those who had already reached LDA at month 6. Indeed, for patients in SDAI LDA at month 6, 68.5 % of patients receiving MTX alone and 88.7 % receiving abatacept plus MTX maintained LDA or achieved remission at month 12 (estimate of difference (95 % CI) 20.2 % (4.4, 36.0)). For patients in SDAI MDA at month 6, 38.8 % of patients receiving MTX alone and 50.8 % receiving abatacept plus MTX achieved LDA or remission by month 12 (estimate of difference (95 % CI) 12.0 % (−5.6, 29.7); see Additional file 5: Figure S2).

### Cumulative probability assessment

**Fig. 2:**
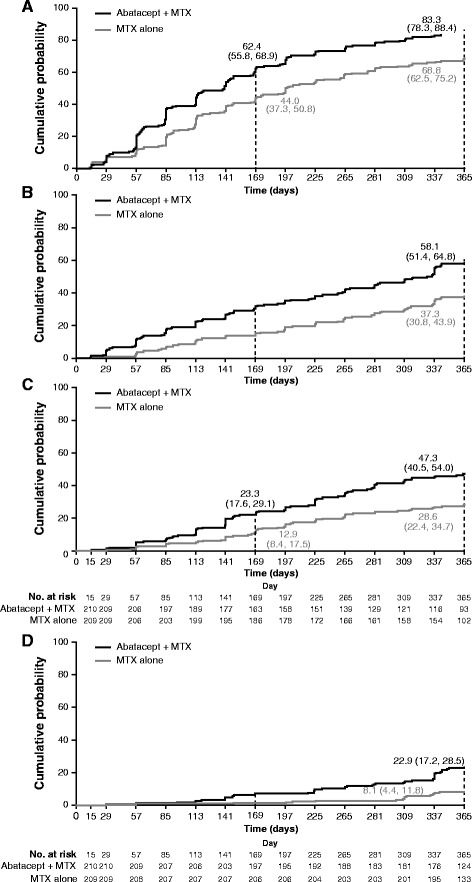
Cumulative probability plot for time to achieve first low disease activity (*LDA*)/remission or sustained LDA/remission. Cumulative probability plot to achieve (**a**) first Simplified Disease Activity Index (*SDAI*) LDA, (**b**) first sustained SDAI LDA, (**c**) first SDAI remission, and (**d**) first sustained SDAI remission over 12 months (Kaplan–Meier estimation), based on patients with data available at baseline, month 6 and month 12. Cumulative probability and corresponding 95 % CI were computed using Kaplan–Meier limit-product estimators. Patients who lost first LDA or remission status were censored at the time of remission loss. SDAI LDA = SDAI ≤11; SDAI remission = SDAI ≤3.3; sustained remission was defined as maintained first LDA or remission at all subsequent visits up to 12 months. Median time to achieve outcome corresponds to the time point when 50 % of patients achieved the outcome. Median time was therefore not calculated if <50 % of patients achieved the outcome over the 12-month period. Median times (95 % CI) to first SDAI LDA and first SDAI remission for the abatacept plus methotrexate (*MTX*) arm were 140.0 and 371.0 days, respectively. Median time (95 % CI) to first SDAI LDA for the MTX arm was 197 days. Median time to achieve first SDAI remission cannot be defined in the MTX arm

Cumulative probability plots to first SDAI LDA and remission and sustained first SDAI LDA and remission over 12 months are shown in Fig. 2 (Kaplan–Meier estimation). Cumulative probability of achieving DAS28 and CDAI remission and LDA within the first 6 months and looking at 12-month outcomes are shown in Table 1. Patients receiving abatacept plus MTX were more likely to achieve first remission or LDA than patients receiving MTX alone, for all criteria. For LDA, all indices gave similar probability of reaching LDA and time to achieve first or sustained LDA. For remission, cumulative probabilities of achieving SDAI or CDAI remission were lower than DAS28, as expected. Although very stringent, regarding the definition of sustained remission, these analyses highlight that a higher proportion of patients receiving abatacept plus MTX attained sustained LDA or remission than patients receiving MTX alone. The median time to achieve first remission was also longer for SDAI and CDAI than for DAS28 (data not shown), indicating greater stringency.

**Table 1:** Cumulative probability (95 % CI) of achieving first LDA/remission or sustained LDA/remission

		First time point at which 95 % CI did not overlap, day	Cumulative probability (95 % CI) of achieving the specified state according to the different indices			
			Month 6		Month 12	
			Abatacept + MTX	MTX alone	Abatacept + MTX	MTX alone
First LDA	DAS28	85	57.6 (50.9, 64.3)	43.5 (36.8, 50.3)	80.6 (75.2, 86.0)	67.6 (61.1, 74.0)
	CDAI	85	60.5 (53.9, 67.1)	43.5 (36.8, 50.3)	83.3 (78.3, 88.4)	69.3 (63.0, 75.6)
First remission	DAS28	57	40.0 (33.4, 46.6)	27.3 (21.3, 33.3)	70.2 (63.9, 76.4)	50.4 (43.5, 57.3)
	CDAI	225	22.9 (17.2, 28.5)	14.4 (9.6, 19.1)	48.4 (41.6, 55.2)	31.2 (24.8, 37.5)
First sustained LDA	DAS28	57	–	–	54.8 (48.0, 61.5)	33.0 (26.6, 39.4)
	CDAI	57	–	–	58.6 (51.9, 65.2)	37.3 (30.8, 43.9)
First sustained remission	DAS28	113	–	–	38.6 (32.0, 45.2)	20.6 (15.1, 26.1)
	CDAI	141	–	–	24.8 (18.9, 30.6)	10.5 (6.4, 14.7)

Based on as-observed analyses for patients with data available at baseline, month 6 and month 12. Cumulative probability evaluated based on Kaplan–Meier estimation with corresponding 95 % CI. First remission/LDA was defined by the first visit when the patient reached remission/LDA according to DAS28 and CDAI scores. Sustained remission/LDA was defined as first remission or LDA status (according to DAS28 and CDAI scores) maintained at all subsequent visits up to month 12; patients who lost remission/LDA status were censored at the time of remission/LDA loss. *CDAI* Clinical Disease Activity Index, *DAS28* Disease Activity Score 28, *LDA* low disease activity, *MTX* methotrexate

### Relationship between DAS28, SDAI and CDAI remission

**Fig. 3:**
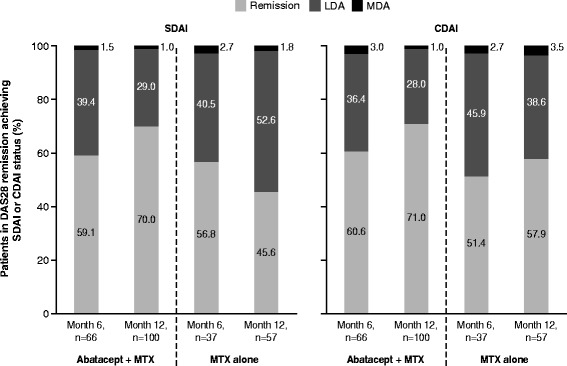
Relationship between Disease Activity Score using 28-joint counts (*DAS28*) remission and Simplified Disease Activity Index (*SDAI*) and Clinical Disease Activity Index (*CDAI*) status, based on patients with data available at baseline, month 6 and month 12 and achieving DAS28 remission at month 6 or 12 (DAS28 remission = DAS28 <2.6); CDAI/SDAI disease activity states are mutually exclusive (a patient can be in only one category at any one time) and were defined as: SDAI remission = SDAI ≤3.3; SDAI low disease activity (*LDA*) = SDAI >3.3–11; SDAI moderate disease activity (*MDA*) = SDAI >11–26; SDAI high disease activity (*HDA*) = SDAI >26; CDAI remission = CDAI ≤3.3; CDAI LDA = CDAI >2.8–10; CDAI MDA = CDAI >10–22; CDAI HDA = CDAI >22. *MTX* methotrexate

Among patients receiving abatacept plus MTX who achieved DAS28 remission, nearly all achieved at least LDA according to SDAI or CDAI (Fig. 3); approximately 60 % and approximately 70 % also achieved SDAI or CDAI remission at months 6 and 12, respectively, suggesting some residual disease activity for patients in DAS28 remission not achieving SDAI or CDAI remission. Few patients (1–4 %) in DAS28 remission were in SDAI- or CDAI-defined MDA, with none in HDA. For patients in SDAI or CDAI remission, nearly all patients (91–100 %) also achieved DAS28 remission at both time points in either treatment arm. With MTX alone, fewer patients in DAS28 remission at month 12 achieved SDAI or CDAI remission compared with those receiving abatacept plus MTX. These data suggest that patients in DAS28 remission receiving abatacept plus MTX may have greater improvements in underlying disease components than patients receiving MTX alone.

### Core set variables

Median values for TJC, SJC, PGA, EGA, CRP and ESR for patients achieving remission were numerically lower for abatacept plus MTX versus MTX alone, irrespective of the index employed (See Additional file 6: Figure S3). The 3rd quartiles (or 75th percentile) of tender and swollen joints for abatacept plus MTX-treated patients in SDAI and CDAI remission were 0 (ranging up to a maximum of 2 for SJC28 and 1 for TJC28), while the median was 1 SJC28 and 0 TJC28 for patients in DAS28 remission (ranging up to a maximum of 6 SJC28 and 3 TJC28). In patients achieving remission status at month 12 but not at month 6, mean clinical core set variables and mean SDAI, CDAI and DAS28 composite scores at baseline and month 6 (Table 2) indicate that, although not yet in remission at month 6, patients experienced major improvements in these variables.

**Table 2:** Clinical outcomes at month 6: patients in remission at month 12 but not month 6

Mean score (SD) (95 % CI)	Patients in SDAI remission at month 12 but not month 6				Patients in CDAI remission at month 12 but not month 6			
	Abatacept + MTX (n = 36)		MTX alone (n = 18)		Abatacept + MTX (n = 37)		MTX alone (n = 23)	
	Baseline	Month 6	Baseline	Month 6	Baseline	Month 6	Baseline	Month 6
DAS28	6.31 (1.01)	3.17 (1.03) (2.82, 3.52)	5.91 (1.10)	3.22 (1.16) (2.64, 3.79)	6.42 (0.97)	3.17 (1.02) (2.83, 3.51)	6.00 (1.07)	3.34 (1.11) (2.86, 3.82)
SDAI	48.73 (16.05)	11.57 (11.29) (7.75, 15.39)	43.71 (14.77)	11.89 (11.81) (6.02, 17.76)	49.67 (15.68)	11.31 (11.22) (7.57, 15.05)	44.58 (14.58)	12.20 (11.03) (7.42, 16.97)
CDAI	45.68 (14.86)	11.07 (11.18) (7.29, 14.85)	41.17 (14.69)	10.79 (11.34) (5.15, 16.43)	46.44 (14.57)	10.76 (11.12) (7.05, 14.47)	41.69 (13.91)	11.05 (10.65) (6.44, 15.65)
TJC28	17.28 (7.23)	4.17 (5.23) (2.40, 5.94)	15.11 (7.41)	4.06 (5.63) (1.26, 6.86)	17.43 (7.37)	3.97 (5.19) (2.24, 5.70)	15.52 (7.29)	4.26 (5.27) (1.98, 6.54)
SJC28	15.25 (6.15)	3.64 (5.16) (1.89, 5.38)	14.11 (5.71)	3.33 (4.98) (0.86, 5.81)	15.38 (6.09)	3.57 (5.10) (1.87, 5.27)	14.35 (5.42)	3.30 (4.68) (1.28, 5.33)
PGA (0–10 cm VAS)	6.57 (2.47)	2.20 (1.48) (1.70, 2.70)	5.66 (2.87)	1.77 (1.25) (1.14, 2.39)	6.92 (2.29)	2.24 (1.50) (1.74, 2.74)	5.67 (2.82)	1.88 (1.30) (1.32, 2.44)
EGA (0–10 cm VAS)	6.59 (2.02)	1.07 (1.09) (0.70, 1.43)	6.28 (2.12)	1.63 (1.22) (1.03, 2.24)	6.71 (1.87)	0.98 (1.07) (0.62, 1.33)	6.15 (1.95)	1.60 (1.15) (1.11, 2.10)
CRP (mg/dL)	3.05 (2.92)	0.50 (0.56) (0.31, 0.69)	2.54 (2.45)	1.10 (1.38) (0.42, 1.78)	3.24 (2.70)	0.56 (0.58) (0.36, 0.75)	2.89 (2.92)	1.15 (1.24) (0.61, 1.68)
ESR (mm/hour)*	47.93 (23.18)	22.60 (15.36) (14.09, 31.11)	39.11 (27.51)	19.22 (16.42) (6.60, 31.85)	49.06 (26.33)	24.94 (20.27) (14.14, 35.74)	46.18 (35.98)	26.36 (22.86) (11.00, 41.72)

*Numbers of patients: n = 15 for abatacept plus MTX and n = 9 for MTX alone for SDAI, and n = 16 for abatacept plus MTX and n = 11 for MTX alone for CDAI; as-observed data for patients with data at baseline, month 6 and month 12. *CDAI* Clinical Disease Activity Index, *CRP* C-reactive protein, *DAS28* Disease Activity Score using 28 joint counts, *EGA* evaluator’s global assessment, *ESR* erythrocyte sedimentation rate, *MTX* methotrexate, *PGA* Patient Global Assessment, *SDAI* Simplified Disease Activity Index, *SJC* swollen joint count, *TJC* tender joint count, *VAS* visual analog scale

### Relationship between disease activity and function

**Fig. 4:**
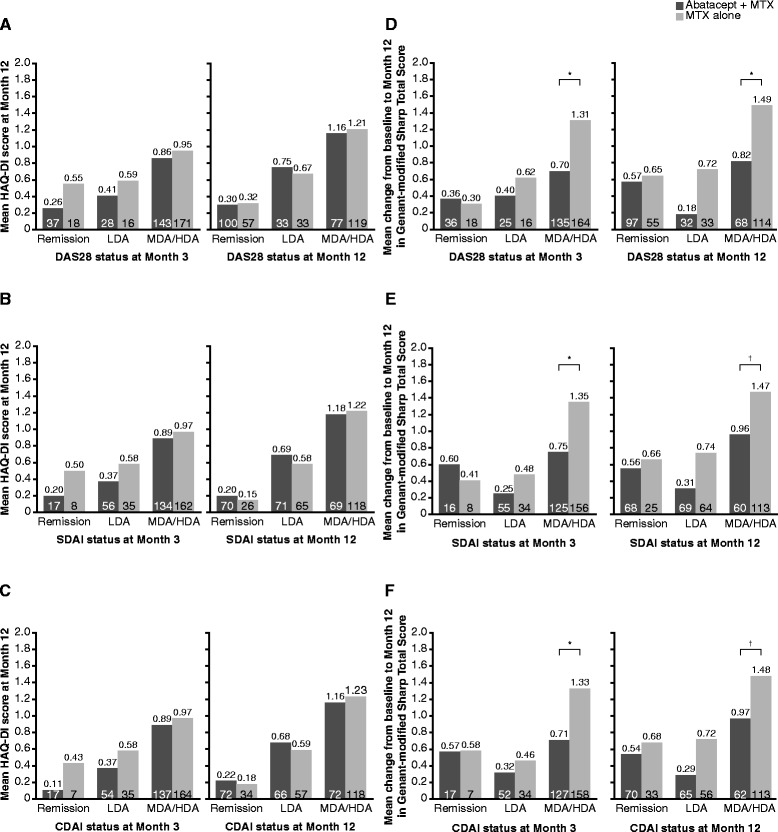
Functional and structural outcomes at month 12 according to disease activity status. Functional (**a**–**c**) and structural (**d**–**f**) outcomes at month 12 according to disease activity status at months 3 and 12, based on patients with data available at baseline, month 6 and month 12. *P <0.05; ^†^Non-significant. Disease activity states are mutually exclusive (a patient can be in only one category at any one time) and were defined as: Disease Activity Score using 28-joint counts (*DAS28*) remission = DAS28 <2.6; DAS28 low disease activity (*LDA*) = DAS28 2.6–3.2; DAS28 moderate disease activity (*MDA*)/high disease activity (*HDA*) = DAS28 >3.2; Simplified Disease Activity Index (*SDAI*) remission = SDAI ≤3.3; SDAI LDA = SDAI >3.3–11; SDAI MDA/HDA = SDAI >11; Clinical Disease Activity Index (*CDAI*) remission = CDAI ≤3.3; CDAI LDA = CDAI >2.8–10; CDAI MDA/HDA = CDAI >10. *ANCOVA* analysis of covariance, *MTX* methotrexate

Patients receiving abatacept plus MTX who achieved remission or LDA according to DAS28, SDAI or CDAI at month 3 achieved greater improvements in HAQ-DI scores at month 12 vs patients with MDA or HDA (Fig. 4a–c). Similar trends were seen for MTX alone, with greater improvements in HAQ-DI scores at month 12 for patients in remission or LDA at month 3 vs patients in MDA or HDA. Similar data were reported at different time points. At all time points, in line with their more stringent nature, mean HAQ-DI scores were numerically lower for patients in SDAI and CDAI remission (with mean HAQ-DI score of ≤0.5 with MTX alone and ≤0.25 with abatacept plus MTX) versus DAS28 remission (see Additional file 7: Table S4).

### Relationship between disease activity and structural outcomes

Structural damage progression assessed by change in TGSS at month 12 was evaluated according to disease activity status at months 3 and 12 (Fig. 4d–f). Patients who achieved remission at months 3 or 12 had only minimal change from baseline in TGSS over 12 months of abatacept plus MTX or MTX alone. However, for patients continuing to be in MDA/HDA at 3 months, estimates of treatment difference and associated exploratory *P* values demonstrated that less structural damage progression was seen in those treated with abatacept plus MTX than in those treated with MTX alone, although progression was not abrogated completely (adjusted mean treatment group differences (95 % CI) were: −0.61 (−1.11, −0.11), *P* <0.05, for patients in DAS28 MDA/HDA at month 3; −0.60 (−1.11, −0.09), *P* <0.05, for patients in SDAI MDA/HDA at month 3; and −0.62 (−1.13, −0.11), *P* <0.05, for patients in CDAI MDA/HDA at month 3; Fig. 4d–f, left panels).

Change from baseline in TGSS at month 12 was also evaluated according to patients’ disease activity status at month 12 (Fig. 4d–f, right panels). Patients treated with abatacept plus MTX who were in MDA/HDA at month 12 had numerically greater changes from baseline compared with patients in LDA or remission. Estimates of treatment difference and associated exploratory *P* values also showed that patients treated with abatacept plus MTX in MDA/HDA tended to have numerically better outcomes in terms of structural damage progression than those treated with MTX alone (adjusted mean treatment group differences (95 % CI) were: −0.68 (−1.33, −0.03), *P* < 0.05, for patients in DAS28 MDA/HDA at month 12; −0.50 (−1.18, 0.18), *P* = 0.146, for patients in SDAI MDA/HDA at month 12; and −0.51 (−1.18, 0.17), *P* = 0.139, for patients in CDAI MDA/HDA at month 12).

## Discussion

In the AGREE study, MTX-naïve patients with early RA who had been treated with abatacept plus MTX experienced clinical, functional and structural benefits, and improvements in disease activity, versus patients treated with MTX alone [15]. In these post hoc analyses, high proportions of patients receiving abatacept plus MTX achieved remission at months 6 and 12 according to the stringent ACR-EULAR index-based SDAI (19 and 33 %, respectively) and CDAI (19 and 34 %, respectively) criteria. Higher proportions of patients achieved remission according to DAS28 (31 and 48 % at months 6 and 12) compared with SDAI, CDAI and Boolean criteria. This was not surprising as it is well established that DAS28 remission criteria frequently are afflicted with significant residual disease activity, both clinically and by sonography [4–9]. Interestingly, the difference between DAS28 remission rates and SDAI/CDAI remission rates was generally smaller here [21–24], presumably because abatacept, unlike some other biologic agents [21], may not directly influence the acute-phase response, which is heavily weighted in the DAS28 formula. However, the DAS28 remission rates observed at 6 months were similar to those previously observed with other biologic agents. Rates for patients achieving Boolean-defined remission (with laboratory measures) were also relatively high for patients treated with abatacept plus MTX (15 and 24 % at months 6 and 12). These findings demonstrate that remission according to the stringent ACR-EULAR remission criteria (SDAI, CDAI and Boolean) is indeed frequently achievable in clinical trials in many MTX-naïve patients treated with MTX, and in even greater proportions of patients receiving biologic therapy (in this case abatacept) in combination with MTX. These results were not dissimilar to those observed in clinical practice, where SDAI and CDAI remission outcomes have been seen in approximately 20–25 % of patients [25].

Analyses of disease activity based on different indices have been performed for other populations of patients treated with abatacept, including those with established RA refractory to MTX [26, 27], and similar to other biologic agents, remission rates are lower for patients with refractory disease treated with abatacept plus MTX/DMARDs than for those in the MTX-naïve population, especially for achieving the stringent SDAI-derived criteria [28, 29].

Cumulative probability plots have shown that patients receiving abatacept plus MTX reached stringent first remission targets at earlier time points than patients receiving MTX alone; this may have an impact on functional and structural outcomes, and so is important to evaluate [2].

As anticipated, not all patients in DAS28 remission also achieved SDAI or CDAI remission, although a large proportion of patients were in SDAI or CDAI LDA, highlighting that it is more difficult to achieve remission according to these stringent criteria or that - in line with the data shown here - DAS28 "remission" comprises many patients with significant residual disease activity which are rightly captured as not being in remission by ACR-EULAR remission criteria and as being in an active disease state by SDAI and CDAI.

For abatacept plus MTX, the proportions of patients achieving stringent remission increased from month 6 to month 12 regardless of the index used, highlighting an increasing magnitude of response over time, even in this RA population with high risk of progression. Such observations, which have not been reported previously with other biologic agents, suggest that the efficacy of abatacept plus MTX may not peak at 6 months, but appears to continue to increase after month 6, allowing more patients to reach the therapeutic goals of SDAI and CDAI remission with continued therapy. These findings are supported by the recently updated EULAR recommendations for the management of RA with DMARDs, which state that individual patients may take longer than 6 months to achieve remission, and that in such cases change in disease activity from baseline should also be taken into consideration [30]. Moreover, these findings highlight the value of the LDA target in the treat-to-target recommendations [31–35] and also support the ACR-EULAR recommendations to report not only achievement but also maintenance of outcomes and sustainability of remission [2].

Finally, we evaluated the association between remission of clinical disease activity at early time points (month 3) with functional and structural outcomes over the 12-month period. Patients who achieved clinical remission demonstrated numerically greater improvements in physical function, especially versus patients in MDA/HDA, regardless of the index used, supporting an association between early suppression of inflammation and long-term functional benefit. The best functional outcomes were achieved with SDAI and CDAI remission. Mean changes in radiographic score were numerically lower for patients in remission or LDA versus MDA/HDA; this difference was less pronounced for patients receiving abatacept plus MTX. Importantly, adjusted mean differences and exploratory *P* values (i.e., not based on prespecified analyses) show a difference between patients in MDA/HDA treated with abatacept plus MTX versus MTX alone, suggesting a dissociation of the profound relationship between inflammation and destruction, as seen previously for tumor necrosis factor inhibitors, tocilizumab and rituximab [31–35]. Altogether, these findings further highlight the value of targeting stringent remission outcomes, as recommended by ACR-EULAR [2].

The limitations of this post hoc descriptive analysis should be taken into consideration when interpreting these data. The population analyzed here represents a subset of the original AGREE study, including only patients with complete datasets. The analyses were neither prespecified nor powered to detect between- or within-group differences. However, only 90/509 randomized patients were excluded from the analysis, which provides a relatively large sample size, and even if those patients excluded had been included and none had achieved remission, the overall proportion of patients achieving remission would likely have still been substantial, especially for patients treated with abatacept plus MTX. Nevertheless, the findings presented here would benefit from validation in a larger population.

## Conclusions

In summary, in contrast to commonly held beliefs [13, 14], relatively high proportions of patients can achieve at least low disease activity or remission by the ACR-EULAR criteria, despite, and at the same time because of, their stringency; this was seen for MTX and was more pronounced for treatment with abatacept plus MTX. In the AGREE study, many patients attained low disease activity, an alternative therapeutic target to remission. Furthermore, the regular assessment of remission in this study may have resulted in patients with transient fluctuations in disease activity failing to achieve the study definition of sustained remission (i.e., maintenance of first remission at all subsequent monthly visits up to month 12), and may have led to an underestimation of sustained remission. Some data suggest that higher rates of remission can be observed in observational datasets and clinical trials when a treat-to-target strategy is adopted [25, 36]. More recent trials of abatacept in MTX-naïve patients with early progressive RA have reported higher rates of remission than reported here, even when using stringent remission criteria [37]. This trend is thought to reflect the increasing use of biologic DMARDs earlier in the course of RA disease, patients entering clinical trials having shorter disease duration and lower disease activity at baseline. Nevertheless, these present analyses support the use of viable treatment targets in the therapy of patients with RA.

## Additional files

Additional file 1:
**Core components comprising disease activity indices.** This table shows the core components and overall cutoff values used to define Disease Activity Score 28 (*DAS28*), Simplified Disease Activity Index (*SDAI*), Clinical Disease Activity Index (*CDAI*) and Boolean remission. Additional file 2:
**Baseline demographics and clinical characteristics.** This table contains the baseline demographics and clinical characteristics for patients with data available at baseline, month 6 and month 12. Additional file 3:
**Baseline demographics and clinical characteristics according to Simplified Disease Activity Index (** ***SDAI*** **) and Disease Activity Score 28 (** ***DAS28*** **) disease activity at month 12.** This table contains the baseline demographics and clinical characteristics for patients with data available at baseline, month 6 and month 12, according to their SDAI and DAS28 disease activity (low, moderate, high, remission) at month 12. Additional file 4:
**Additional disease activity outcomes.** This file contains a multi-part figure. **A** Boolean remission rates at month 6 and month 12 in the abatacept plus methotrexate (*MTX*) and MTX alone treatment groups, comparing three definitions of Boolean remission (three criteria (28 joints), all criteria (66/68 joints), three criteria (66/68 joints)). **B** Rates of low disease activity (*LDA*) at months 6 and 12, comparing three criteria for assessing LDA (Disease Activity Score 28 (*DAS28*), Simplified Disease Activity Index (*SDAI*) and Clinical Disease Activity Index (*CDAI*)). Additional file 5:
**Shifts in Simplified Disease Activity Index (** ***SDAI*** **) category from month 6 to month 12.** This figure shows the change in SDAI disease activity (low (*LDA*), moderate (*MDA*), high (*HDA*), or remission) between month 6 and month 12 in the abatacept plus methotrexate (*MTX*) and MTX alone treatment groups. Additional file 6:
**Core set variables for patients in remission.** This table contains a multi-part figure comparing core set variables at month 6 in patients who achieve Disease Activity Score 28 (*DAS28*), Clinical Disease Activity Index (*CDAI*) or Simplified Disease Activity Index (*SDAI*) remission. Figure A shows patient and physician assessment of global disease activity, Figure B shows swollen and tender joint counts, and Figure C shows C-reactive protein level and erythrocyte sedimentation rate. Additional file 7:
**Functional outcomes according to the Simplified Disease Activity Index (** ***SDAI*** **).** This table shows the mean (standard deviation) physical function, as assessed by Health Assessment Questionnaire-Disability Index (*HAQ-DI*) score, according to SDAI disease activity state (low, moderate/high, remission) at months 3, 6 and 12.

## Abbreviations

ACR: American College of Rheumatology
AGREE: Abatacept study to Gauge Remission and joint damage progression in methotrexate (MTX)-naive patients with Early erosive rheumatoid arthritis
CDAI: Clinical Disease Activity Index
CRP: C-reactive protein
DAS28: Disease Activity Score 28
DMARD: Disease-modifying antirheumatic drug
EGA: evaluator’s global assessment
ESR: erythrocyte sedimentation rate
EULAR: European League Against Rheumatism
HAQ-DI: Health Assessment Questionnaire-Disability Index
HDA: high disease activity
LDA: low disease activity
MDA: moderate disease activity
MTX: methotrexate
PGA: patient global assessment
RA: rheumatoid arthritis
SDAI: Simplified Disease Activity Index
SJC: swollen joint count
TJC: tender joint count
TGSS: total Genant-modified Sharp score
