# Supplementary material for: Attainment and characteristics of clinical remission according to the new ACR-EULAR criteria in abatacept-treated patients with early rheumatoid arthritis: new analyses from the Abatacept study to Gauge Remission and joint damage progression in methotrexate (MTX)-naive patients with Early Erosive rheumatoid arthritis (AGREE)
Source: Arthritis Res Ther. 2015 Jun 11;17(1):157. doi: 10.1186/s13075-015-0671-9 (PMC4494702; doi:10.1186/s13075-015-0671-9)
Supplement: Additional file 1: — Core components comprising disease activity indices. This table shows the core components and overall cutoff values used to define Disease Activity Score 28 (DAS28), Simplified Disease Activity Index (SDAI), Clinical Disease Activity Index (CDAI) and Boolean remission. [file 13075_2015_671_MOESM1_ESM.docx]

**Supplementary Table 1 Core components comprising disease activity indices**

|  | **DAS28** | **SDAI** | **CDAI** | **Boolean** | |
| --- | --- | --- | --- | --- | --- |
|  |  |  |  | **Including laboratory measures** | **Excluding laboratory measures** |
| TJC28 | ✓ | ✓ | ✓ | ✓ | ✓ |
| SJC28 | ✓ | ✓ | ✓ | ✓ | ✓ |
| PGA* | ✓ | ✓ | ✓ | ✓ | ✓ |
| EGA |  | ✓ | ✓ |  |  |
| CRP^†^ | ✓ | ✓ |  | ✓ |  |
| Remission cut-off | <2.6 | ≤3.3 | ≤2.8 | All component scores ≤1 | |

*Based on 0–100 mm VAS for DAS28, and on 0–10 cm VAS for SDAI, CDAI or Boolean criteria; ^†^in mg/L for DAS28 and in mg/dL for SDAI and Boolean remission. CDAI = Clinical Disease Activity Index; CRP = C-reactive protein; DAS28 = Disease Activity Score using 28 joint counts; EGA = evaluator’s global assessment; PGA = patient global assessment; SDAI = Simplified Disease Activity Index; SJC = swollen joint count; TJC = tender joint count; VAS = visual analog scale.
