# Supplementary material for: Attainment and characteristics of clinical remission according to the new ACR-EULAR criteria in abatacept-treated patients with early rheumatoid arthritis: new analyses from the Abatacept study to Gauge Remission and joint damage progression in methotrexate (MTX)-naive patients with Early Erosive rheumatoid arthritis (AGREE)
Source: Arthritis Res Ther. 2015 Jun 11;17(1):157. doi: 10.1186/s13075-015-0671-9 (PMC4494702; doi:10.1186/s13075-015-0671-9)
Supplement: Additional file 2: — Baseline demographics and clinical characteristics. This table contains the baseline demographics and clinical characteristics for patients with data available at baseline, month 6 and month 12. [file 13075_2015_671_MOESM2_ESM.docx]

**Supplementary Table 2 Baseline demographics and clinical characteristics**

|  | **Abatacept + MTX (n=210)** | **MTX alone**  **(n=209)** |
| --- | --- | --- |
| Age, years | 49.4 (12.5) | 49.2 (12.8) |
| Female, % | 75.2 | 80.4 |
| Disease duration, months | 6.2 (7.5) | 7.1 (7.2) |
| Tender joints (66/68 count) | 31.3 (14.9) | 30.9 (14.2) |
| Swollen joints (66/68 count) | 22.7 (11.3) | 22.4 (10.6) |
| RF positive, % | 96.7 | 97.1 |
| ACPA positive, % | 93.3 | 87.6 |
| RF and ACPA positive, % | 90.5 | 85.2 |
| CRP level, mg/dL | 3.2 (3.1) | 3.7 (5.3) |
| DAS28 (CRP) | 6.3 (1.0) | 6.3 (1.0) |
| SDAI | 48.9 (15.2) | 48.2 (14.9) |
| CDAI | 45.6 (14.0) | 44.5 (13.1) |
| HAQ-DI | 1.7 (0.7) | 1.7 (0.7) |
| Total x-ray score | 7.7 (9.6) | 6.8 (8.8) |
| Subject pain assessment, 100-mm VAS | 66.6 (23.3) | 66.8 (23.4) |
| Patient global assessment, 100-mm VAS | 65.8 (22.3) | 63.3 (24.5) |
| Physician global assessment, 100-mm VAS | 67.6 (18.4) | 64.9 (19.2) |

Data are based on patients with data available at baseline, Month 6 and Month 12. Data are mean (SD) unless stated otherwise. ACPA = anti-cyclic citrullinated peptide antibody; CDAI = Clinical Disease Activity Index; CRP = C-reactive protein; DAS28 = disease activity score using 28 joint counts; HAQ-DI = Health Assessment Questionnaire-Disability Index; MTX = methotrexate; RF = rheumatoid factor; SD = standard deviation; SDAI = Simplified Disease Activity Index; VAS = visual analog scale.
