# Supplementary material for: Attainment and characteristics of clinical remission according to the new ACR-EULAR criteria in abatacept-treated patients with early rheumatoid arthritis: new analyses from the Abatacept study to Gauge Remission and joint damage progression in methotrexate (MTX)-naive patients with Early Erosive rheumatoid arthritis (AGREE)
Source: Arthritis Res Ther. 2015 Jun 11;17(1):157. doi: 10.1186/s13075-015-0671-9 (PMC4494702; doi:10.1186/s13075-015-0671-9)
Supplement: Additional file 3: — Baseline demographics and clinical characteristics according to Simplified Disease Activity Index ( SDAI ) and Disease Activity Score 28 ( DAS28 ) disease activity at month 12. This table contains the baseline demographics and clinical characteristics for patients with data available at baseline, month 6 and month 12, according to their SDAI and DAS28 disease activity (low, moderate, high, remission) at month 12. [file 13075_2015_671_MOESM3_ESM.docx]

**Supplementary Table 3 Baseline demographics and clinical characteristics according to SDAI and DAS28 disease activity at Month 12**

| **Baseline values** | **Abatacept + MTX (n=210)** | | | | **MTX alone (n=209)** | | | |
| --- | --- | --- | --- | --- | --- | --- | --- | --- |
|  | **SDAI and DAS28 disease activity at Month 12** | | | | **SDAI and DAS28 disease activity at Month 12** | | | |
|  | **SDAI remission (n=70)** | **SDAI LDA (n=71)** | **SDAI MDA (n=49)** | **SDAI HDA (n=20)** | **SDAI remission (n=26)** | **SDAI LDA (n=65)** | **SDAI MDA (n=70)** | **SDAI HDA (n=48)** |
| Disease duration, months | 5.5 (6.4) | 5.2 (6.5) | 7.1 (8.4) | 10.1 (10.6) | 6.5 (7.3) | 7.4 (7.1) | 7.4 (7.5) | 6.6 (6.9) |
| TJC28 | 15.9 (6.9) | 16.6 (6.7) | 18.4 (6.8) | 22.3 (6.3) | 15.8 (6.6) | 16.6 (6.5) | 16.2 (6.8) | 19.2 (6.9) |
| SJC28 | 14.3 (6.0) | 14.4 (5.6) | 15.4 (6.1) | 18.7 (6.5) | 14.3 (5.5) | 13.9 (5.0) | 14.2 (5.3) | 16.5 (6.4) |
| RF positive, % | 98.6 | 93.0 | 100 | 95.0 | 96.2 | 100 | 97.1 | 93.8 |
| ACPA positive, % | 94.3 | 97.2 | 91.8 | 80.0 | 92.3 | 80.0 | 91.4 | 89.6 |
| DAS28 (CRP) | 6.1 (1.0) | 6.3 (0.9) | 6.4 (0.9) | 7.0 (0.9) | 6.0 (1.0) | 6.2 (1.0) | 6.2 (1.0) | 6.6 (1.0) |
| SDAI | 45.9 (15.1) | 47.7 (14.3) | 50.5 (14.7) | 59.5 (15.1) | 44.8 (14.1) | 46.6 (13.8) | 46.7 (13.6) | 54.3 (17.1) |
| CDAI | 43.1 (14.4) | 44.4 (13.2) | 47.4 (13.4) | 54.7 (13.8) | 42.5 (14.1) | 42.8 (12.0) | 43.9 (12.5) | 48.6 (14.1) |
| HAQ-DI | 1.6 (0.7) | 1.7 (0.6) | 1.8 (0.6) | 2.1 (0.7) | 1.5 (0.7) | 1.5 (0.7) | 1.7 (0.6) | 1.9 (0.7) |
|  | **DAS28 remission (n=100)** | **DAS28 LDA (n=33)** | **DAS28 MDA (n=60)** | **DAS28 HDA (n=17)** | **DAS28 remission (n=57)** | **DAS28 LDA (n=33)** | **DAS28 MDA (n=87)** | **DAS28 HDA (n=32)** |
| Disease duration, months | 5.8 (6.7) | 4.1 (5.3) | 6.8 (8.0) | 10.8 (11.2) | 6.4 (6.9) | 7.1 (6.5) | 7.4 (7.6) | 7.7 (7.4) |
| TJC28 | 16.0 (7.0) | 16.7 (6.4) | 18.4 (6.8) | 22.9 (5.8) | 16.1 (6.4) | 17.8 (6.3) | 16.1 (7.0) | 20.0 (6.6) |
| SJC28 | 14.3 (5.7) | 14.5 (5.7) | 15.5 (6.3) | 18.3 (6.8) | 14.0 (5.6) | 15.5 (4.6) | 13.9 (5.4) | 17.0 (6.4) |
| RF positive, % | 98.0 | 90.9 | 98.3 | 94.1 | 98.2 | 100 | 95.4 | 96.9 |
| ACPA positive, % | 96.0 | 93.9 | 90.0 | 88.2 | 87.7 | 72.7 | 92.0 | 90.6 |
| DAS28 (CRP) | 6.2 (1.0) | 6.3 (1.0) | 6.4 (0.9) | 7.1 (0.8) | 6.1 (0.9) | 6.2 (1.2) | 6.2 (1.0) | 6.8 (0.9) |
| SDAI | 46.5 (14.5) | 48.0 (14.9) | 50.1 (15.5) | 60.0 (13.8) | 46.0 (14.2) | 49.0 (14.3) | 46.0 (13.3) | 57.2 (17.7) |
| CDAI | 43.3 (13.7) | 45.0 (13.4) | 47.1 (14.1) | 55.3 (13.1) | 42.8 (12.9) | 45.7 (12.4) | 42.9 (12.8) | 50.5 (13.5) |
| HAQ-DI | 1.6 (0.7) | 1.7 (0.6) | 1.8 (0.6) | 2.1 (0.6) | 1.5 (0.7) | 1.5 (0.8) | 1.7 (0.6) | 2.0 (0.6) |

Data are based on patients with data available at baseline, Month 6 and Month 12. Data are mean (SD) unless stated otherwise.

Disease activity states are mutually exclusive (a patient can be in only one category at any one time) and were defined as: DAS28 remission = DAS28 <2.6; DAS28 LDA = DAS28 2.6–3.2; DAS28 MDA = DAS28 >3.2–5.1; DAS28 HDA = DAS28 >5.1; SDAI remission = SDAI ≤3.3; SDAI LDA = SDAI >3.3–11; SDAI MDA = SDAI >11–26; SDAI HDA = SDAI >26. ACPA = anti-cyclic citrullinated peptide antibody; CDAI = Clinical Disease Activity Index; CRP = C-reactive protein; DAS28 = disease activity score using 28 joint counts; HAQ-DI = Health Assessment Questionnaire-Disability Index; HDA = high disease activity; LDA = low disease activity; MDA = moderate disease activity; MTX = methotrexate; RF = rheumatoid factor; SD = standard deviation; SDAI = Simplified Disease Activity Index; SJC = swollen joint count; TJC = tender joint count.
