# Supplementary material for: Attainment and characteristics of clinical remission according to the new ACR-EULAR criteria in abatacept-treated patients with early rheumatoid arthritis: new analyses from the Abatacept study to Gauge Remission and joint damage progression in methotrexate (MTX)-naive patients with Early Erosive rheumatoid arthritis (AGREE)
Source: Arthritis Res Ther. 2015 Jun 11;17(1):157. doi: 10.1186/s13075-015-0671-9 (PMC4494702; doi:10.1186/s13075-015-0671-9)

Supplementary Figure 1 Additional disease activity outcomes.
A. Boolean remission rates. Based on patients with data available at baseline, Month 6 and Month 12. **B.** LDA rates at Months 6 and 12 for DAS28, SDAI and CDAI. DAS28 LDA = DAS28 ≤3.2; SDAI LDA = SDAI ≤11; CDAI LDA = CDAI ≤10. Boolean remission definitions according to three criteria (28 joints) = TJC28 ≤1 and SJC28 ≤1 and PGA (0–10 cm VAS) ≤1 cm; all criteria (66/68 joints) = TJC66/68 ≤1 and SJC66/68 ≤1 and CRP ≤1 mg/dL and PGA ≤1 cm; and three criteria (66/68 joints) = TJC66/68 ≤1 and SJC66/68 ≤1 and PGA ≤1 cm. CDAI = Clinical Disease Activity Index; CI = confidence interval; CRP = C-reactive protein; DAS28 = disease activity score using 28 joint counts; MTX = methotrexate; LDA = low disease activity; PGA = patient global assessment; SDAI = Simplified Disease Activity Index; SJC = swollen joint count; TJC = tender joint count.

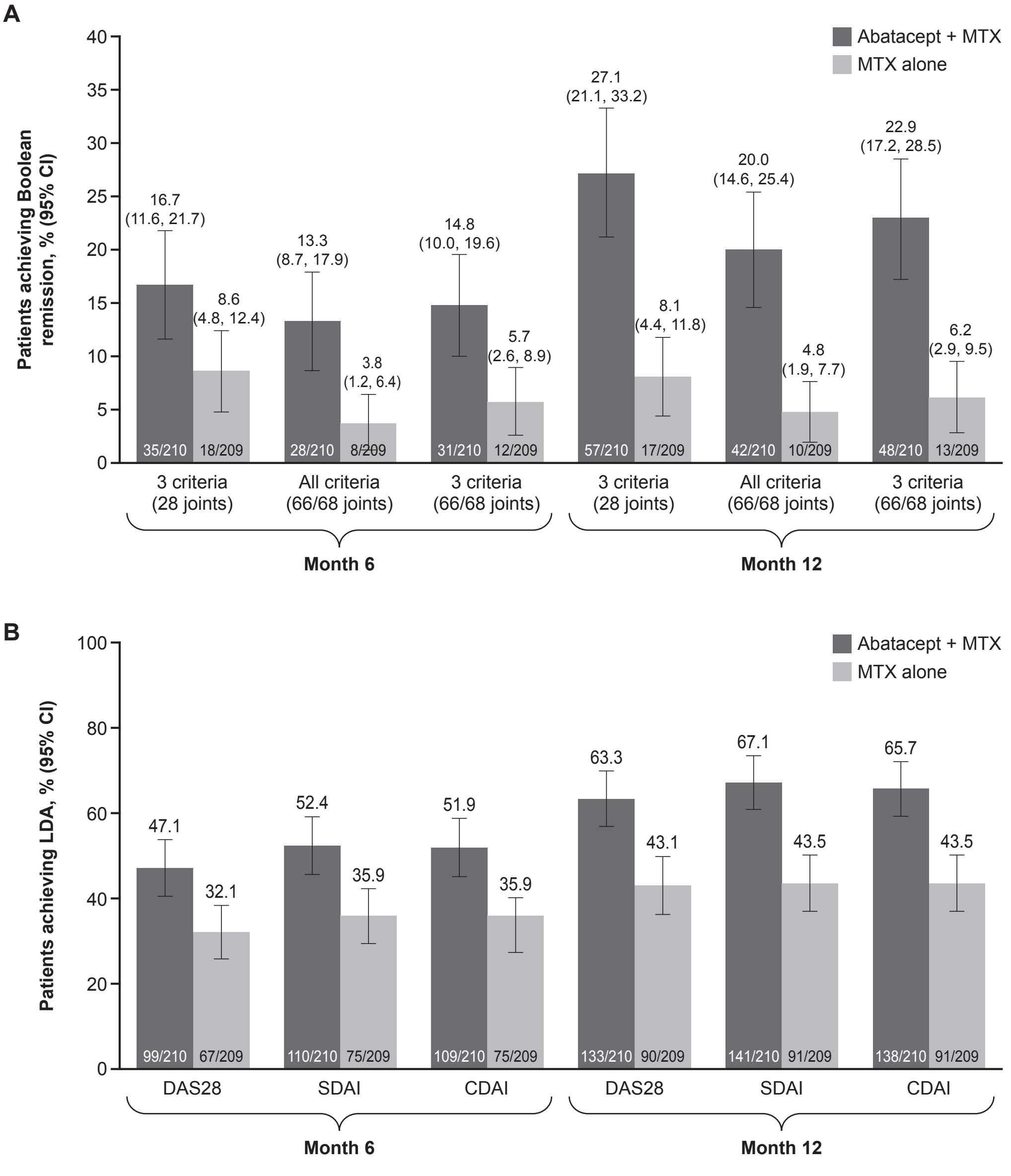

Supplement: Additional file 4: — Additional disease activity outcomes. This file contains a multi-part figure. A Boolean remission rates at month 6 and month 12 in the abatacept plus methotrexate (MTX) and MTX alone treatment groups, comparing three definitions of Boolean remission (three criteria (28 joints), all criteria (66/68 joints), three criteria (66/68 joints)). B Rates of low disease activity (LDA) at months 6 and 12, comparing three criteria for assessing LDA (Disease Activity Score 28 (DAS28), Simplified Disease Activity Index (SDAI) and Clinical Disease Activity Index (CDAI)). [file 13075_2015_671_MOESM4_ESM.pdf]
