# Supplementary material for: Attainment and characteristics of clinical remission according to the new ACR-EULAR criteria in abatacept-treated patients with early rheumatoid arthritis: new analyses from the Abatacept study to Gauge Remission and joint damage progression in methotrexate (MTX)-naive patients with Early Erosive rheumatoid arthritis (AGREE)
Source: Arthritis Res Ther. 2015 Jun 11;17(1):157. doi: 10.1186/s13075-015-0671-9 (PMC4494702; doi:10.1186/s13075-015-0671-9)

Supplementary Figure 2 Shifts in SDAI disease activity category from Month 6 to Month 12.

Based on patients with data available at baseline, Month 6 and Month 12. SDAI disease activity states are mutually exclusive (a patient can be in only one category at any one time) and were defined as: remission = SDAI ≤3.3; LDA = SDAI >3.3–11; MDA = SDAI >11–26; HDA = SDAI >26. HDA = high disease activity; LDA = low disease activity; MDA = moderate disease activity; MTX = methotrexate; SDAI = Simplified Disease Activity Index.

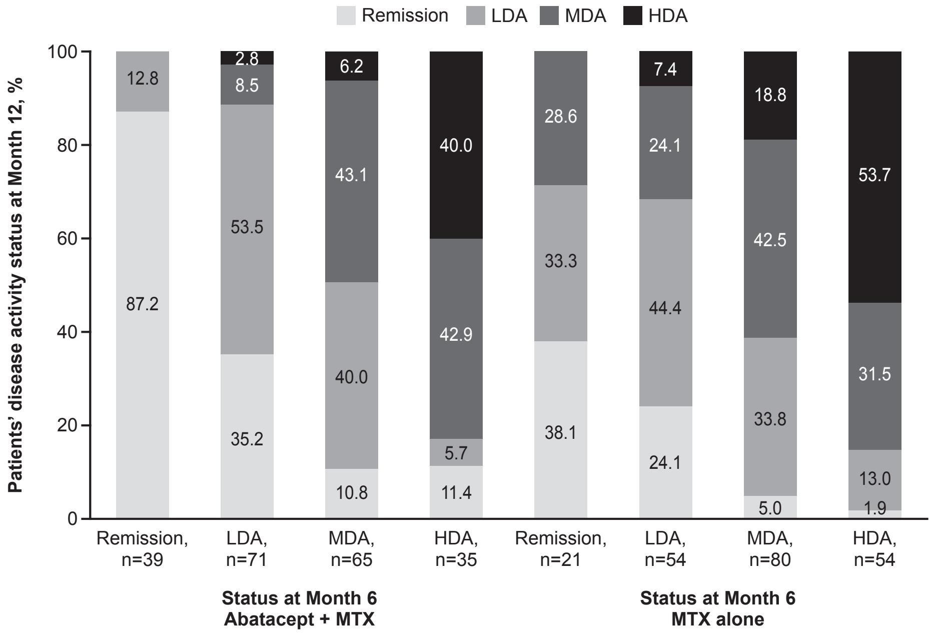

Supplement: Additional file 5: — Shifts in Simplified Disease Activity Index ( SDAI ) category from month 6 to month 12. This figure shows the change in SDAI disease activity (low (LDA), moderate (MDA), high (HDA), or remission) between month 6 and month 12 in the abatacept plus methotrexate (MTX) and MTX alone treatment groups. [file 13075_2015_671_MOESM5_ESM.pdf]
