# Supplementary material for: Attainment and characteristics of clinical remission according to the new ACR-EULAR criteria in abatacept-treated patients with early rheumatoid arthritis: new analyses from the Abatacept study to Gauge Remission and joint damage progression in methotrexate (MTX)-naive patients with Early Erosive rheumatoid arthritis (AGREE)
Source: Arthritis Res Ther. 2015 Jun 11;17(1):157. doi: 10.1186/s13075-015-0671-9 (PMC4494702; doi:10.1186/s13075-015-0671-9)
Supplement: Additional file 6: — Core set variables for patients in remission. This table contains a multi-part figure comparing core set variables at month 6 in patients who achieve Disease Activity Score 28 (DAS28), Clinical Disease Activity Index (CDAI) or Simplified Disease Activity Index (SDAI) remission. Figure A shows patient and physician assessment of global disease activity, Figure B shows swollen and tender joint counts, and Figure C shows C-reactive protein level and erythrocyte sedimentation rate. [file 13075_2015_671_MOESM6_ESM.pdf]

**Supplementary Figure 3  Core set variables for patients in remission.**
Data are based on patients with data available at baseline, Month 6 and Month 12. Data presented are min, Q1, median, Q3 and max values from bottom to top of the box plot, respectively. Dotted line indicates ACR-EULAR Boolean remission criteria cut-off of ≤1 for SJC, TJC, PGA and CRP (mg/dL). ACR = American College of Rheumatology; DAS28 remission = DAS28 (CRP) <2.6; SDAI remission = SDAI ≤3.3; CDAI remission = ≤2.8. CDAI = Clinical Disease Activity Index; CRP = C-reactive protein; DAS28 = disease activity score using 28 joint counts; EGA = evaluator’s global assessment; EULAR = European League Against Rheumatism; MTX = methotrexate; PGA = patient global assessment; SDAI = Simplified Disease Activity Index; SJC = swollen joint count; TJC = tender joint count; VAS = visual analog scale.

A

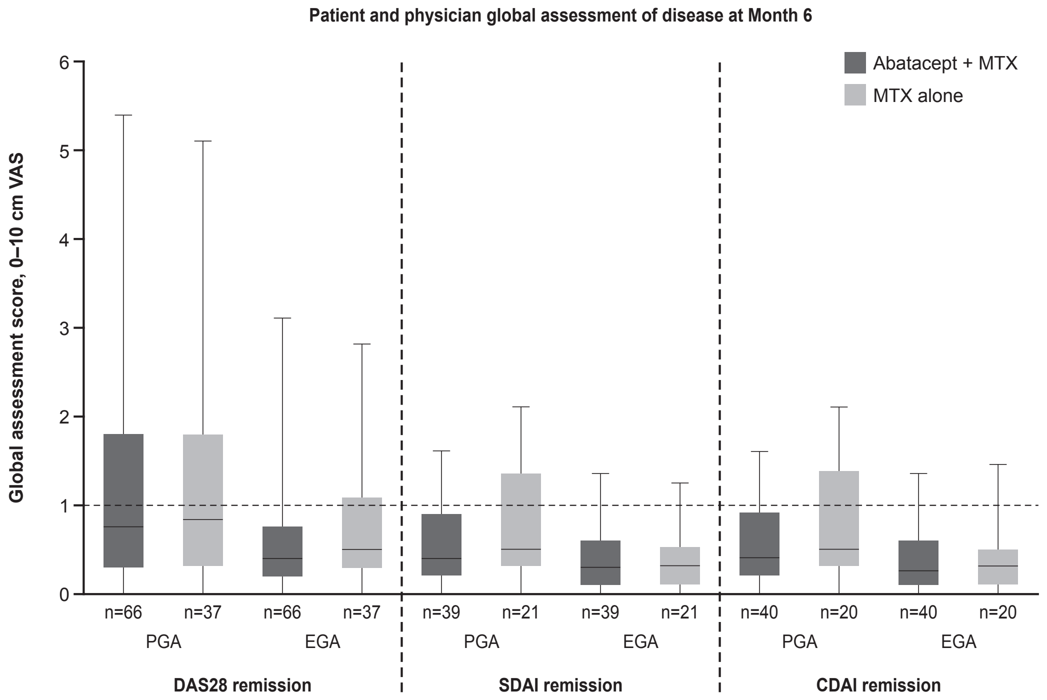

**B**

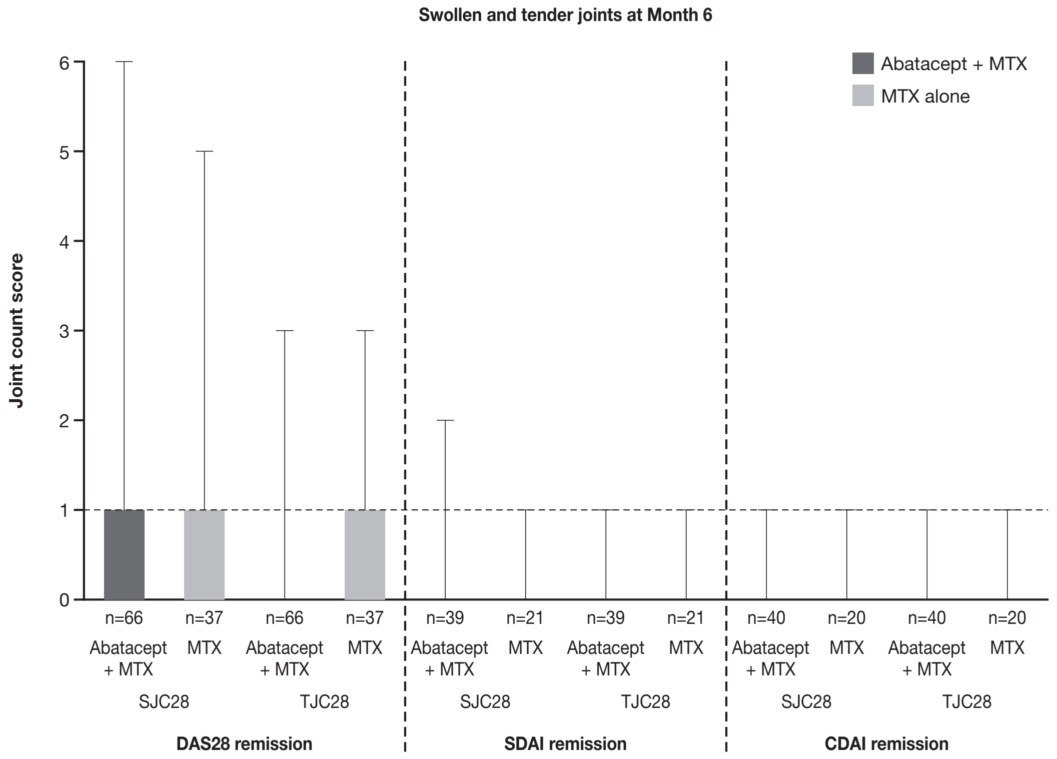

Swollen and tender joints at Month 6
Abatacept + MTX
MTX alone
Joint count score
0
1
2
3
4
5
6
n=66
n=37
n=66
n=37
n=39
n=21
n=39
n=21
n=40
n=20
n=40
n=20
Abatacept + MTX
MTX
SJC28
TJC28
DAS28 remission
SDAI remission
CDAI remission


**C**

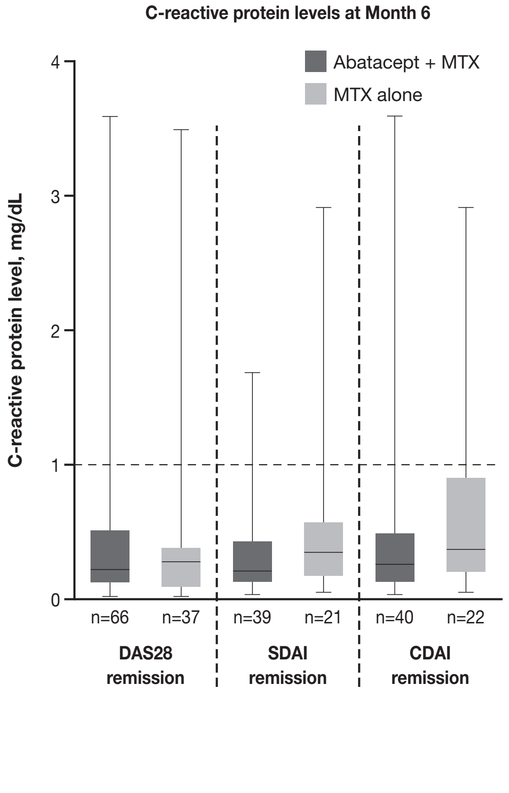

C-reactive protein levels at Month 6
Abatacept + MTX
MTX alone
C-reactive protein level, mg/dL
0
1
2
3
4
n=66
n=37
n=39
n=21
n=40
n=22
DAS28 remission
SDAI remission
CDAI remission


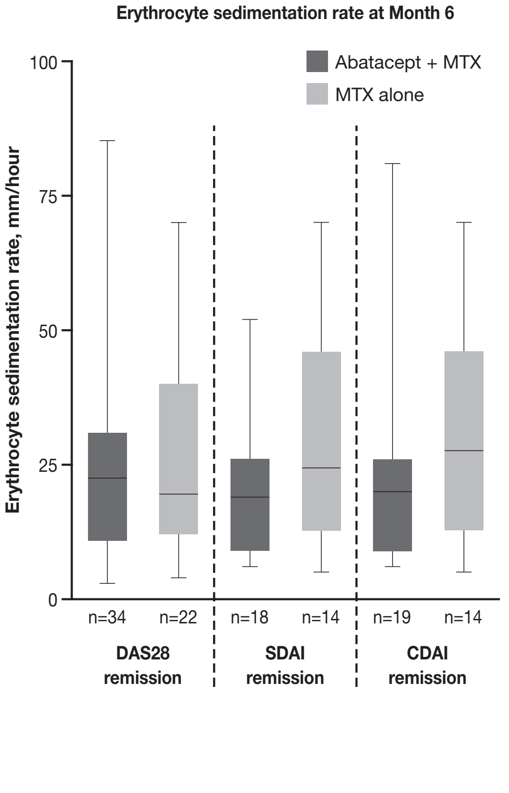

Erythrocyte sedimentation rate at Month 6
Abatacept + MTX
MTX alone
Erythrocyte sedimentation rate, mm/hour
0
25
50
75
100
n=34
n=22
n=18
n=14
n=19
n=14
DAS28 remission
SDAI remission
CDAI remission
