# Supplementary material for: Attainment and characteristics of clinical remission according to the new ACR-EULAR criteria in abatacept-treated patients with early rheumatoid arthritis: new analyses from the Abatacept study to Gauge Remission and joint damage progression in methotrexate (MTX)-naive patients with Early Erosive rheumatoid arthritis (AGREE)
Source: Arthritis Res Ther. 2015 Jun 11;17(1):157. doi: 10.1186/s13075-015-0671-9 (PMC4494702; doi:10.1186/s13075-015-0671-9)
Supplement: Additional file 7: — Functional outcomes according to the Simplified Disease Activity Index ( SDAI ). This table shows the mean (standard deviation) physical function, as assessed by Health Assessment Questionnaire-Disability Index (HAQ-DI) score, according to SDAI disease activity state (low, moderate/high, remission) at months 3, 6 and 12. [file 13075_2015_671_MOESM7_ESM.docx]

**Supplementary Table 4 Functional outcomes according to SDAI disease state**

| **Mean score (SD)** | | **Abatacept + MTX** | | | | **MTX alone** | | | |
| --- | --- | --- | --- | --- | --- | --- | --- | --- | --- |
|  |  | **n** | **Month 3** | **Month 6** | **Month 12** | **n** | **Month 3** | **Month 6** | **Month 12** |
| SDAI state at Month 3 | Remission | 17 | 0.26 (0.35) | 0.18 (0.30) | 0.20 (0.31) | 8 | 0.42 (0.48) | 0.27 (0.36) | 0.50 (0.61) |
|  | LDA | 56 | 0.57 (0.46) | 0.48 (0.49) | 0.37 (0.49) | 35 | 0.71 (0.53) | 0.56 (0.51) | 0.58 (0.60) |
|  | MDA/HDA | 134 | 1.19 (0.65) | 1.02 (0.73) | 0.89 (0.69) | 162 | 1.26 (0.63) | 1.09 (0.66) | 0.97 (0.69) |
| SDAI state at Month 6 | Remission | 39 | NA | 0.16 (0.21) | 0.12 (0.23) | 21 | NA | 0.37 (0.37) | 0.43 (0.53) |
|  | LDA | 71 | NA | 0.69 (0.57) | 0.55 (0.59) | 54 | NA | 0.62 (0.55) | 0.60 (0.64) |
|  | MDA/HDA | 100 | NA | 1.13 (0.74) | 1.00 (0.66) | 134 | NA | 1.20 (0.62) | 1.07 (0.66) |
| SDAI state at Month 12 | Remission | 70 | NA | NA | 0.20 (0.34) | 26 | NA | NA | 0.15 (0.22) |
|  | LDA | 71 | NA | NA | 0.69 (0.56) | 65 | NA | NA | 0.58 (0.57) |
|  | MDA/HDA | 69 | NA | NA | 1.18 (0.66) | 118 | NA | NA | 1.22 (0.61) |

Remission = SDAI ≤3.3; LDA = SDAI >3.3–11; MDA = SDAI >11–26; HDA = SDAI >26. HDA = high disease activity; LDA = low disease activity; MDA = moderate disease activity; MTX = methotrexate; NA = not available; SD = standard deviation; SDAI = Simplified Disease Activity Index.
